# Climate change and dengue: a critical and systematic review of quantitative modelling approaches

**DOI:** 10.1186/1471-2334-14-167

**Published:** 2014-03-26

**Authors:** Suchithra Naish, Pat Dale, John S Mackenzie, John McBride, Kerrie Mengersen, Shilu Tong

**Affiliations:** 1 School of Public Health and Social Work & Institute of Health and Biomedical Innovation, Queensland University of Technology, Victoria Park Road, Brisbane, Queensland, Australia; 2 Environmental Futures Centre, Australian Rivers Institute, Griffith School of Environment Griffith University, Brisbane, Queensland, Australia; 3 Faculty of Health Sciences, Curtin University, Perth, Australia; 4 School of Medicine and Dentistry, James Cook University, Cairns, Queensland, Australia; 5 Mathematical Sciences, Statistical Science, Queensland University of Technology, George Street, Brisbane, Queensland, Australia

**Keywords:** Climate, Dengue, Models, Projection, Scenarios

## Abstract

**Background:**

Many studies have found associations between climatic conditions and dengue transmission. However, there is a debate about the future impacts of climate change on dengue transmission. This paper reviewed epidemiological evidence on the relationship between climate and dengue with a focus on quantitative methods for assessing the potential impacts of climate change on global dengue transmission.

**Methods:**

A literature search was conducted in October 2012, using the electronic databases PubMed, Scopus, ScienceDirect, ProQuest, and Web of Science. The search focused on peer-reviewed journal articles published in English from January 1991 through October 2012.

**Results:**

Sixteen studies met the inclusion criteria and most studies showed that the transmission of dengue is highly sensitive to climatic conditions, especially temperature, rainfall and relative humidity. Studies on the potential impacts of climate change on dengue indicate increased climatic suitability for transmission and an expansion of the geographic regions at risk during this century. A variety of quantitative modelling approaches were used in the studies. Several key methodological issues and current knowledge gaps were identified through this review.

**Conclusions:**

It is important to assemble spatio-temporal patterns of dengue transmission compatible with long-term data on climate and other socio-ecological changes and this would advance projections of dengue risks associated with climate change.

## Background

Dengue is a major public health concern for over half of the world’s population and is a leading cause of hospitalisation and death, particularly for children in endemic countries [1]. Recent studies estimate that 3.6 billion people are at risk, with over 230 million infections, millions of cases with dengue fever, over 2 million cases with severe disease, and 21,000 deaths [1-5]. A 30-fold increase in the number of dengue cases over the past 50 years has been recorded with nearly 119 countries endemic for dengue [4]. As part of global climate changes, temperature has increased by a global average of 0.75°C over the past 100 years. Temperature increases such as these are potentially associated with substantial increases in dengue outbreaks. Apart from climate factors other important issues that potentially contribute to global changes in dengue incidence and distribution include population growth, urbanisation, lack of sanitation, increased human travel, ineffective mosquito control, and increased reporting capacity [3-9].

Dengue is primarily transmitted by *Aedes aegypti* and secondarily by *Aedes albopictus*. Both mosquitoes have adapted to local human habitation with oviposition and larval habitats in natural (e.g., rock pools, tree holes and leaf axis) and artificial (e.g., water tanks, blocked drains, pot plants and food and beverage containers) collections in the urban and peri-urban environment. These mosquitoes may be infected with any of the four dengue viruses with an incubation period of 3–14 days [10]. Dengue affects people of all ages, including infants irrespective of gender. Dengue viruses cause a spectrum of disease, with symptoms from mild influenza-like symptoms to severe or fatal haemorrhage fever [11].

### The ecology of dengue and vector

The epidemiological triangle of dengue includes host, pathogen and mosquito vectors (including *Ae. aegypti* and *Ae. Albopictus)* together with their interactions in the environment. Dengue is climate sensitive as the virus has to complete part of its development in the mosquito vectors that transmit the disease [5]. The major vector is *Ae. aegypti* whose life cycle is directly influenced by ambient temperature and rainfall [12]. Increased temperature could increase dengue risk by increasing the rate of mosquito development and reducing virus incubation time in areas where the vector presently exists, thereby increasing the rate of transmission [13-16]. Conversely, extreme hot temperatures may also increase the rate of mosquito mortality and thus decrease dengue risk [17]. Similarly, rainfall can have non-linear contrasting effects on dengue risk [6,17]. Heavy rainfall may flush away eggs, larvae, and pupae from containers in the short term but residual water can create breeding habitats in the longer term [18]. A dry climate can lead to human behaviour of saving water in water storage containers, which may become breeding sites for *Ae. Aegypti*[19]*.* Thus, climatic conditions may affect the virus, the vector and/or human behaviour both directly and indirectly [20]. Studies have demonstrated that the ecology of virus is intrinsically tied to the ecology of dengue vectors [21]. Recent studies have also elaborated on the impacts of climate change on the vector, for example, how the extreme climatic events drive mosquito outbreaks [22-25]. However, empirical relationships have been demonstrated between climate variables, dengue and dengue vectors, casual relationships have not been strongly established.

### Dengue and climate change

It is established that climate change is happening and it is likely to expand the geographical distribution of several mosquito-borne diseases [26]. The mounting evidence around climate-disease relationships raises many important issues about the potential effects of global climate changes on the transmission of infectious diseases, particularly dengue [5,27-29]. There is evidence indicating that dengue epidemics have been associated with temperature [30-32], rainfall [33,34] and relative humidity [35-37]. Few studies have included spatial data in climate-based predictive models [33,38].

As global climate change is predicted to accelerate over the next a few decades at least [1,3-6,27], an increased frequency, intensity and duration of extreme climatic events are more likely, so affecting dengue transmission. This is a global public health priority. A better understanding of the relationship between climate and disease is an important step towards finding ways to mitigate the impact of disease on communities, for example, malaria [39]. Successful future management of dengue requires an understanding of the dynamics of the virus, host, vector, and environmental factors especially in the context of a changing climate [21].

### Quantitative modelling of dengue and climate

The influences drawn about the relationships between dengue and climate, and the predictions of dengue under future climate change scenarios may depend on the analytical approaches used. The aim of this paper is to review the relevant literature on dengue disease and climate with a focus on quantitative models of the impact of climate change; to address methodological issues in this challenging field and then to indicate future opportunities and research directions.

## Methods

### Search strategy

**Figure 1:**
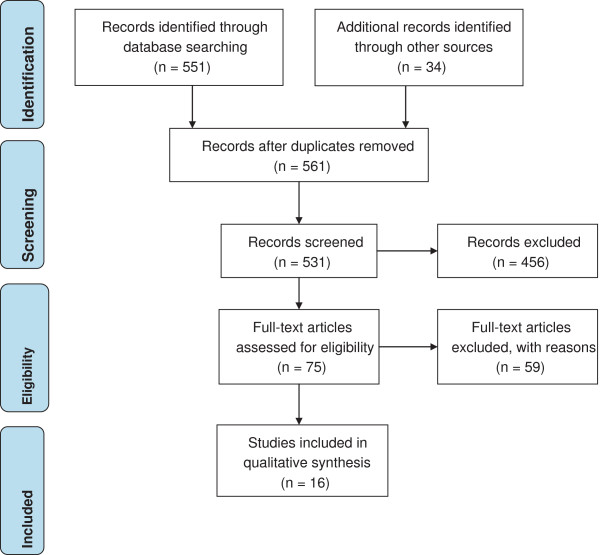
Flowchart of literature search.

A literature search was conducted in October 2012 using the electronic databases PubMed, Scopus and ScienceDirect, ProQuest and Web of Science to obtain the information on the impact of climate variables (and climate change) on dengue disease transmission. The search period included January 1991 (the commencement of the reporting of dengue in Queensland, Australia) to October 2012. We limited the literature search to journal articles published in English, available in full-text/ pdf. The key words used were dengue disease transmission, climate and/or climate change, projection /forecast and scenario but not diagnostic tests, vector/ virus type. References and citations of the articles identified were checked to ensure that all relevant articles were included (Figure 1).

### Selection criteria

Three selection criteria were used to select articles from the search results for the detailed consideration. First, in order to obtain authoritative information, this review included only peer-reviewed journal articles. Second, articles had to include larger geographical areas, climate data including climate parameters and statistical analytical methods and at least one climate-based projection of future dengue disease transmission. Finally, we included only quantitative studies based on statistical models (climate-based) because qualitative studies used an entirely different research design and analytic approach.

We assessed the strengths and limitations of analytical models and their use of established relationship between climate change and dengue transmission. Finally, we provide recommendations for future research directions towards model parameters and predictions of climate change on dengue transmission.

## Results

The initial search yielded 531 studies of which 456 were deemed to be potentially relevant and were subjected to further perusal. This led to 75 studies considered in detail and 16 that strictly met the inclusion criteria. Table 1 shows the characteristics of the 16 studies that addressed the climate variables (climate change) and future risk of dengue transmission, using climate change scenarios.

**Table 1:** Studies included in the review of climate impact on dengue

**References**	**Study area (Period)**	**Dengue data**	**Covariate data**	**Spatial resolution**	**Analytical approaches**	**Key findings**	**Comments**
Earnest et al. [32]	Singapore (2001–2008)	Weekly laboratory confirmed notified dengue cases	Weekly climate (mean/minimum/maximum temperature, mean rainfall, mean/minimum/maximum relative humidity, mean hours of sunshine and mean hours of cloud) data	Local meteorological station data	Poisson regression, Sinusoidal function	Temperature, relative humidity and SOI associated with dengue cases.	Temporal trends of dengue were noticeable.
Descloux et al. [31]	Noumea (New Caledonia) (1971–2010)	Monthly confirmed cases of DF/ DHF	Monthly climate (temperature, precipitation, relative humidity, wind force, potential evapo-transpiration, hydric balance sheet) data and ENSO indices	Local meteorological station data	Non-linear models	Significant inter-annual correlations were observed between dengue outbreaks and summertime temperature, precipitation, relative humidity but not ENSO.	The epidemic dynamics of dengue were driven by climate.
Chen et al. [30]	Taiwan (1994–2008)	Daily confirmed cases of notified DF	Daily climate (temperature, rainfall) data, socio-demographic factors	Local meteorological station data	GAM	Rainfall was correlated with dengue cases. Lag effects were observed.	A climatic change does have influence on dengue outbreaks.
Hu et al. [43]	Australia (2002–2005)	Monthly confirmed cases of notified dengue	Monthly weather, SEIFA, pop (LGA)	Local meteorological station data	Bayesian CAR	Increase in dengue cases of 6% in association with a 1-mm increase in average monthly rainfall and a 1°C increase in average monthly maximum temperature, respectively was observed.	Socio-ecological factors appear to influence dengue. The drivers may differ for local and overseas cases. Spatial clustering of dengue cases was evident.
Chowell [45]	Peru (1994–2008)	Annual confirmed cases	Time series of annual population size and density, altitude and climate data	Local meteorological station data	Wavelet time series	A significant difference in the timing of epidemics between jungle and coastal regions was observed.	The differences in the timing of dengue epidemics between jungle and coastal regions were significantly associated with the timing of the seasonal temperature cycle.
Thai et al. [50]	Vietnam (1994–2009)	Monthly confirmed cases	Monthly climate (mean temperature, rainfall and relative humidity) data and ENSO indices	Local meteorological station data	Wavelet time series	ENSO indices and climate variables were significantly associated with dengue incidence.	Climate variability and ENSO impact dengue outbreaks.
Colon-Gonzalez et al. [41]	Mexico (1985–2007)	Monthly confirmed cases	Monthly climate (minimum and maximum temperature and rainfall) and ENSO indices	Local meteorological station data	Linear regression, Phillips–Perron and Jarque–Bera test tests	Incidence was higher during El-Nino. Incidence was associated with El-Nino and temperature during cool and dry times.	Temperature was an important factor in the dengue incidence in Mexico.
Pinto et al. [9]	Singapore (2000–2007)	Weekly confirmed notified DF cases	Weekly climate (maximum and minimum temperature, maximum and minimum relative humidity) data	Local meteorological station data	Poisson regression, Principal component anlaysis	For every 2–10 degrees of maximum and minimum temperature variation, an increase of cases of 22-184% and 26-230% respectively, was observed.	Temperature was the best predictor for the dengue increase in Singapore.
Gharbi et al. [36]	French West Indies (2000–2007)	Weekly laboratory confirmed cases from hospitals or not	Weekly climate (cumulative rainfall, relative humidity, minimum, maximum and average temperature) data	Local meteorological station data	Time series (SARIMA), RMSE and Wilcoxon signed-ranks test	Temperature was significantly associated with dengue forecasting but not humidity.	Temperature improves dengue outbreaks better than humidity and rainfall.
Hu et al. [42]	Australia (1993–2005)	Monthly confirmed cases of notified DF cases	Monthly SOI, rainfall and annual population	Local meteorological station data	Cross-correlations, Time series (SARIMA)	A decrease in the SOI was significantly associated with an increase in the dengue cases.	Climate variability is directly and/or indirectly associated with dengue. SOI based epidemic forecasting is possible.
Johansson et al. [48]	Puerto Rico, Mexico, Thailand (1986–2006)	Monthly reported cases of DF/ DHF	Monthly climate (precipitation, minimum, maximum and mean average temperature) data and ENSO indices	Global climate surfaces (0.5 × 0.5°) local meteorological station data	Wavelet time series	Temperature, rainfall and dengue incidence were strongly associated in all three countries for the annual cycle. The associations with ENSO varied between countries in the multi-annual cycle.	The role of ENSO may be obscured by local climate heterogeneity, insufficient data, randomly coincident outbreaks, and other, potentially stronger, intrinsic factors regulating dengue transmission dynamics.
Bambrick et al. [44]	Australia (1991–2007)	Annual incidence – notified cases of DF	Annual Temperature, vapour pressure and population	Local meteorological station data	Climate change scenarios	Geographic regions with climates that are favourable to dengue could expand to include large population centres in a number of currently dengue-free regions.	An eight-fold increase in the number of people living in dengue prone regions in Australia will occur unless greenhouses gases are reduced.
Bulto et al. [46]	Cuba (1961–1990)	Dengue-specific parameters of DF/ DHF	Monthly climate (maximum and minimum temperature, precipitation, atmospheric pressure, vapour pressure, relative humidity, thermal oscillation and solar radiation) data	Local meteorological station data	Multivariate (Empiric orthogonal function)	Strong associations between climate anomalies and dengue	Climate variability has influence on dengue.
Cazelles et al. [40]	Thailand (1983–1997)	Monthly confirmed cases of DHF	Monthly climate (temperature and rainfall) data and ENSO indices	Local meteorological station data	Wavelet time series	Strong association between dengue incidence and El-Nino events was observed. Temperature had greater influence on dengue than rainfall.	The association is non-stationary and have a major influence on the synchrony of dengue epidemics.
Hales et al. [33]	Global (1975–1996)	Monthly reported cases of DF	Monthly climate (maximum, minimum and mean temperature, rainfall and vapour pressure) and population and projections (future climate and population) data	Region-specific and GCM projections	Logistic regression and IPCC scenarios	In 2085, under climate and population projections, 50-60% of the population would be at dengue risk.	There is a potential increase in the dengue risk areas under climate change scenarios, if the risk factors remain constant.
Patz et al. [38]	Global (1931–1980)	Dengue-specific parameters	Monthly climate data	Site-specific GCM	GCM output to vectorial capacity	Among the three GCMs, the average projected temperature elevation was 1.16°C, expected by the year 2050.	Epidemic potential increased with a relatively small temperature rise, indicating that lower mosquitoes infestation values would be necessary to maintain or spread dengue in a vulnerable population.

### Dengue data

For the purpose of this study, we have considered Dengue Fever (DF) or Dengue Hemorrhagic Fever (DHF) as a single entity ‘dengue’ as such kind of information was not available from many studies. Eight studies used monthly confirmed and reported dengue notified case data [8,31,33,34,40-43], two studies used annual cases [44,45], three studies used weekly confirmed case data [9,32,36] aggregated from daily surveillance and one study used daily cases [30]. Two studies combined dengue-specific (entomological) parameters [38,46]. Most of the studies used dengue laboratory-confirmed case data (notified) obtained from health departments (Table 1) and some used reported cases [36,47,48].

### Covariate data

Most data were aggregated to monthly estimates from daily, weekly and annual data obtained from meteorological stations. With the exception of a few studies that used global gridded projection data sets [34,38], all other studies obtained climate variables from local/ national meteorological stations [31,32,36,40-43,45,49,50]. Data on socio-economic heterogeneity, climatic diversity including both tropical and subtropical, and un-observed confounders such as social behaviour were described in individual studies.

### Analytical approaches

Among the selected studies, six used SARIMA- time series or wavelet time series models [34,36,40,42,45,50], four used different types of regression analysis [9,32,33,41] and other studies have variously employed a general additive mixed (GAM) model [30], a spatial model [43], a non-linear model [31], a multivariate model [46], and global circulation models (GCM) towards projections [33,38]. Most studies used a combination of the different analytical models but to varying degrees. For example, among studies that used regression analysis, some introduced autoregressive terms. A few studies used Poisson regression models to allow for autocorrelation and over dispersion. A number of studies incorporated the probability of risk of seasonal forecasts as covariates in their analysis [31,36] and only few studies demonstrated the potential use of forecasting in the development of climate-driven models [33,38,44]. The details of these models are explained below.

#### Linear regression models

Colon-Gonzalez et al. [41] used multiple linear regression models to examine the associations between changes in the climate variability and dengue incidence in the warm and humid regions of Mexico for the years 1985–2007. Their results showed that the incidence was higher during El- Niño events and in the warm and wet season. Their study demonstrated that dengue incidence was positively associated with the strength of El-Niño and the minimum temperature, especially during the cool and dry season.

#### Time series/wavelet time series models

Time series modelling approaches have been extensively applied in assessing the impact of climate variables on dengue incidence. For example, Gharbi et al. [36] fitted a seasonal autoregressive integrated moving average (SARIMA) model of dengue incidence and climate variables including temperature, rainfall and relative humidity in French West Indies for the period 2000–2006. They found that temperature significantly improved the ability of the model to forecast dengue incidence but this was not so for humidity and rainfall. They also found that minimum temperature at 5 weeks lag time was the best climatic variable for predicting dengue outbreaks. Similarly, Hu et al. [42] used a time series SARIMA model to examine the impact of El-Niño on dengue in Queensland, Australia for the period 1993–2005. They suggested that a lower Southern Oscillation Index (SOI) was related to increased dengue cases.

Wavelet time series analysis has been applied to examine the associations between El-Niño Southern Oscillation (ENSO), local weather, and dengue incidence in Puerto Rico, Mexico, and Thailand [34] particularly, with the aim of identifying time- and frequency-specific associations. In all three countries, temperature, rainfall, and dengue incidence were strongly associated on an annual scale. On a multiyear scale, ENSO was associated with temperature and with dengue incidence in Puerto Rico, but only for part of the study period. Only local rainfall was associated with the incidence of dengue in that country. The lack of a direct association between ENSO and weather variables to dengue incidence suggests that the ENSO-dengue association may be a spurious result. In Thailand, ENSO was associated with both temperature and rainfall, and rainfall was associated with dengue incidence. However, detailed analysis suggested that this latter association was also probably spurious. The authors concluded that there was no significant association between any of the variables in Mexico on the multiyear scale. In another study, Cazelles et al. [40] used a wavelet time series analysis to demonstrate a strong non-stationary association between dengue incidence and El-Niño in Thailand for the years 1986 to 1992. They suggested that under certain conditions, interannual variation in local or regional climate linked to El-Niño may determine the temporal and spatial dynamics of dengue. Thai et al. [50] used a wavelet time series analysis to investigate the associations between climate variables including mean temperature, humidity and rainfall, and ENSO indices and dengue incidence in Vietnam during the period 1994 to 2009. Their results showed that the ENSO indices and climate variables were significantly associated with dengue incidence in the 2 to 3-year periodic band, although the associations were transient in time. Chowell [45] used wavelet time series analysis to determine the relationship between climatic factors including mean, maximum and minimum temperature and rainfall and dengue incidence for the period 1994–2008 in jungle and coastal regions of Peru. They revealed that incidence was highly associated with seasonal temperature and suggested that dengue was frequently imported into coastal regions through infective sparks from endemic jungle areas and/or cities of other neighbouring endemic countries.

#### Poisson regression models

Poisson regression models have been applied in determining the relationship between climate and dengue. For example, Earnest et al. [32] used this approach to determine the association between climate variables (temperature, humidity, rainfall), ENSO indices and dengue in Singapore. They found that temperature, relative humidity and ENSO were significantly and independently associated with dengue cases. No one set of climate variables was superior to the others, so they suggested that all the climate variables had a similar predictive ability.

Pinto et al. [9] used Poisson regression model to determine the impact of climate variables (temperature, rainfall and relative humidity) on dengue cases in Singapore. They found that for every 2-10°C of variation of the maximum temperature, dengue cases were increased by 22.2-184.6%. For the minimum temperature, for the same variation, they observed that there was an average increase of 26.1-230.3% in the number of the dengue cases from April to August. Their study concluded that the variable temperature (maximum and minimum) was the best predictor for the increased number of dengue cases.

Chen et al. [49] applied Poisson regression using a GAM model to examine the relationship between precipitation and dengue in Taiwan for the period 1994–2008. The GAM allows a Poisson regression to be fit as a sum of nonparametric smooth functions of predictor variables. They found that differential lag effects following precipitation were statistically associated with increased risk of dengue. Poisson regression, using a GAM model was used to evaluate the multiple-lag effects of stratified precipitation levels on specific diseases. All models were adjusted for the multiple-lag effects of daily temperature, month, and township for evaluating the associations between categorized extreme precipitation and diseases, with further trend tests performed to examine linear associations between levels of precipitation and outbreaks of each disease.

#### Bayesian models

Bayesian spatial conditional autoregressive modelling approaches have been used to demonstrate the impact of climatic, social and ecological factors on dengue in Queensland, Australia [43]. The authors suggested that 6% increase in locally acquired dengue was observed in association with a 1-mm increase in average monthly rainfall and a 1°C increase in average monthly maximum temperature. They also reported that overseas-acquired dengue cases were increased by 1% in association with a 1-mm increase in average monthly rainfall and a 1-unit increase in average socioeconomic index, respectively.

#### Non-linear models

Descloux et al. [31] developed an early warning system using a long-term data set (39 years) including dengue cases and meteorological data (mean temp, min and max temperature, relative humidity, precipitation and ENSO indices) in New Caledonia, using multivariate non-linear models. They observed a strong seasonality of dengue epidemics and found significant inter-annual correlations between epidemics and temperature, precipitation and relative humidity. Bulto et al. [46] applied empiric orthogonal function (EOF) to estimate the association between climate data including monthly maximum and minimum mean temperatures, precipitation, atmospheric pressure, vapour pressure, relative humidity, thermal oscillation, days with precipitation, solar radiation in, and isolation and dengue in Cuba during the period 1961–1990. The EOF is designed to obtain the dominant variability patterns from sets of fields of any type, synthetic indicators or indexes, and summarise the variability observed in a group of variables. Climatic anomalies included were multivariate ENSO index, quasi-biennial oscillation and North Atlantic Oscillation. They found a strong association between climate anomalies and dengue which demonstrated significant climate variability.

In summary, the quantitative models employed for evaluating the relationship between climate variables and dengue have been typically different with respect to the distributional assumptions (e.g., normal, Poisson), the nature of the relationship (linear and non-linear) and the spatial and/or temporal dynamics of the response. Overall, the models consistently reveal variability in the relationship between dengue and climate variables, related to country, but the methods identified an association with temperature (except for [49]) followed by rainfall in the majority of research.

#### Projections of climate change impacts on dengue

Patz et al. [38] examined the potential risk posed by global climate change on dengue transmission. They used vectorial capacity equation that was modified to estimate the epidemics of dengue. The model used project climate change data from global circulation model (GCM) at 250 km x 250 km resolution project future risk of dengue globally. Their findings suggest that increased incidences have predominantly occurred in regions bordering endemic zones in latitude or altitude. They found that epidemic activity increased with a small rise in temperature, indicating that fewer mosquitoes would be necessary to maintain or spread dengue in a population at risk of dengue. They concluded that transmission may be saturated in hyper-endemic tropical regions and human migration patterns of susceptible individuals are likely to be more important to overall transmission than are climatic factors. Endemic locations may be at higher risk from dengue if transmission intensity increases. Hales et al. [33] estimated the changes in the geographical limits of dengue transmission from 1975 to 1996 and the size of population at risk, using logistic regression. Monthly averages of vapour pressure, rainfall, and temperature recorded between 1961 and 1990, with or without statistical interaction terms between variables were included in their statistical models. Based on data from GCM projections and human demographics, they predicted that dengue would increase and include a larger total population and higher percent of the population. Although their studies suggested that the dengue distribution was climate dependent, other factors needed to be considered in addition to climate during epidemics.

Bambrick et al. [44] highlighted the potential for climate change to affect the safety and supply of blood globally through its impact on vector-borne disease, using the example of dengue in Australia as a case-study. They modelled geographic regions that were suitable for dengue transmission over the coming century under four climate change scenarios, estimated changes to the population at risk and effect on blood supply. They applied logistic regression models using climate change scenarios to the observed geographic distribution of dengue in Australia. The most important predictor variables in the models were vapour pressure and temperature. Their results indicated that geographic regions with climates that are favourable to dengue transmission could expand to include large population centres in a number of currently dengue-free regions in Australia.

### Methodological issues

Several methodological issues emerged when we conducted this literature review on climate change and dengue. These issues include the study design, analytical model, time period, scale of analysis, exposure variables and other factors associated with dengue transmission as illustrated below.

#### Study designs

Several study designs have been considered by various authors while studying the relationships between climate and dengue (Table 1). For example, Hales et al. [47] used a mixed ecological study design and long-term data to determine the relationship between the annual number of dengue cases, ENSO, temperature and rainfall using global atmospheric analyses climate-based data. Descloux et al. [31] used long-term observational data and examined inter-annual correlations between ENSO, local climate and dengue.

#### Analytical models

Time series modelling approaches have been applied to estimate the baseline relationships between climate and dengue [9,36,40,42,50,51] (Table 1). SARIMA models are potentially useful when there are time dependences between each observation [36,52]. The assumption that each observation is correlated to previous ones makes it possible to model a temporal structure, with more reliable predictions, especially for climate-sensitive diseases (e.g., mosquito-borne diseases), than those obtained by other statistical methods. SARIMA models have been successfully used in epidemiology to predict the evolution of infectious diseases. Moreover, these models allow the integration of external factors, such as climatic variables, that may increase the predictive power and robustness of predictive models due to longer time periods of data.

Some studies have used Fourier analysis to analyse relationships between oscillating time series. This technique decomposes time series into their periodic components that can then be compared between time series. Since Fourier analysis cannot take into account temporal changes in the periodic behaviour of time series (i.e. non-stationarity), this method, and others such as generalized linear models, may be inadequate for investigating the determinants of transmission dynamics of dengue [53].

Wavelet analysis is suitable for investigating time series data from non-stationary systems and for inferring associations between such systems [53]. This approach reveals how the different scales (i.e. the periodic components) of the time series change over time. Wavelet analysis is able to measure associations between two time series at any period. Wavelet analyses have been used to compare time series of disease incidence across localities and countries for the characterisation of the evolution of epidemic periodicity and the identification of synchrony. Wavelet analyses have been used in analysing dengue [34,40,50]. Although annual periodic patterns are a common phenomenon in dengue endemic areas, the identification of a periodic multi-annual (e.g., 2 to 3 years) cycle differs between countries as well as in analyses used. Cazelles et al. [40] used wavelet approaches to demonstrate a highly significant but discontinuous association between ENSO, precipitation and dengue epidemics in Thailand.

A continuous annual mode of oscillation with a non-stationary 2 to 3-year multi-annual cycle was found with strong irregular associations between dengue incidence and ENSO indices and climate variables in Vietnam [50]. Although these wavelet analyses have provided important contribution to the cyclical dynamics of dengue transmission, the associations with ENSO have been irregular and temporary, which reduce the potential for estimating future predictions based on these climate anomalies.

#### Time period

Choosing a baseline time period for climate data is also important. Climate and dengue relationships in the same city can be very different between the 1960s and the 2010s. Differences could be due to socioeconomic and, demographic changes and urbanisation. Differences in the time periods used to estimate the historical climate and dengue relationships also make it difficult to compare projections across studies. Therefore, it has been recommended that long-term (generally, at least many decades) baseline climate data be used for modelling climate-based diseases to calculate an average that is not influenced by climate variability [54,55].

#### Spatial and temporal scales

Another issue to be considered when modelling is the spatio-temporal scale of analysis. This is because spatial and temporal characteristics may provide useful information on risk assessments to be used by local or national dengue prevention and control programs to prepare for and respond to dengue epidemics in endemic settings. Dengue may be sensitive to differences in climatic conditions at a local, regional or global level. At the global level, there is the potential for climate-related spread of dengue. There are areas that are currently only at risk and not endemic for dengue, but may become endemic as climate changes, especially related to temperature change. For example, Patz et al. [38] used a process-based model considering an entomological variable, i.e., the vectorial capacity (VC). They included temperature effects over VC and predicted the potential global dengue spread using climate change scenarios for 2050. Therefore, we suggest to develop models at a local and/or regional scale that can increase their predictive capacity [56] and incorporate important local factors that affect disease transmission [54].

#### Climate variables

The choice of climate variables in the modelling is an important issue to consider. The following climate variables have been considered in the studies: maximum, minimum and mean temperature, rainfall, relative humidity, ENSO indices and sunshine. Among these, temperature and ENSO indices have been found to be important in any study. Authors have identified that other variables such as river levels are also included along with climate variables [57], however, these studies are out of the study scope. Hales et al. [33] developed an empirical model fitting logistic regression and modelled dengue outbreak (presence/absence) considering climate baseline data for the period 1961–1990. They found that the vapour pressure was the only explanatory climate variable responsible for global dengue transmission. Using this model, applying climate projections for 2085 from a GCM, Hales et al. predicted a limited extension of dengue. In both the projection studies, climate projections were based on historical exposure-response functions of climate variables and dengue incidence that are applied to climate change models and emissions scenarios to estimate and predict future dengue distribution. Therefore, it is important to consider which climate variables are the best predictors of dengue transmission and the research reported here indicates that temperature is consistently important but that vapour pressure or relative humidity are also significant contributors.

#### Other factors associated with dengue transmission

There are other environmental, socio-economic and demographic factors associated with dengue transmission and the relative contribution of these factors may differ between settings (scales, countries, regions). These include, but are not limited to attenuators (e.g., mosquito management, screening dwellings, using personal insect repellents and bed nets) or exacerbators (e.g., actively storing of water in open containers, passively allowing water to remain in bins, garden accoutrements) [58]. These are outside the scope of our current review. However, socio-demographic change is important and we have briefly considered this below.

#### Associations between socio-demographic changes and dengue

Other etiologic factors that impact dengue transmission include social and demographic changes, economic status, human behaviour and education [7,43,59-61]. Reiter et al. [62] evidenced that dengue virus transmission was limited by human life style in Texas. Other important human related factors such as exceptional population growth, unplanned urbanisation and air travel needs to be considered in modelling studies [63]. Other factors driven by economic growth such as animals and commodities should also be considered. For example, Gubler et al. [63] claimed that a dramatic urban growth has occurred in the past 40 years providing the suitable ecological conditions for *Ae. aegypti* to increase in close association with large and crowded human populations in tropical areas, creating ideal conditions for dengue transmission.

Dengue-infected human movement should be considered as another important factor [64] since *Aedes* generally has a short flight range so is unlikely to spread dengue over large distances. For example, Rabbai et al. [65] have demonstrated dengue viral exchange between the urban areas of Ho Chi Minh City and other provinces of southern Vietnam and suggested that human movement between urban and rural areas may play a key role in the transmission of dengue virus across southern Vietnam.

### Challenges

The fundamental challenge for predicting dengue transmission is how to best model future climate at a regional and/or local level. In another words, how can we appropriately downscale the GCM modelling outcomes to a regional and/or local level? The Intergovernmental Panel on Climate Change (IPCC) has developed 40 Special Reports on Emissions Scenarios (SRES) covering a wide range of main driving forces of future green house gas emissions [26]. These scenarios were categorised into four classes: A1, A2, B1 and B2. A1 characterises rapid economic growth, population growth by 2050, introduction of new and efficient technologies. A2 characterises high population growth, slow economic development and technological changes. B1 characterises the similar population growth like A1 but with rapid economic changes. B2 characterises medium population and economic growth with localised solutions to economic, social and environmental sustainability. These scenarios can be used to project future climate based on GCM models [26].

Selecting climate models is not a trivial task, considering the strengths and limitations of various GCM models. The IPCC recommended that no single GCM can be considered the best and that various GCMs should be applied to account for climate modelling uncertainties [26]. These do not necessarily indicate errors in the modelling and should therefore not be used to conclude that a model is inaccurate. As the global temperature increases, tropical insects may spread their habitats into more northern or southern latitudes which can result in higher transmission. This intriguing idea was first suggested by Shope [12] and was considered further by other researchers [15,66]. However, there is still debate whether the increased pattern of dengue arose from climate change [16] or from socio-economic changes in combination with ecologic and demographic changes [63,67]. Finally, other etiological factors must ultimately be incorporated into integrated modelling to determine human risk to dengue [68].

## Discussion

Climatic factors play a significant role in the mosquito biology, the viruses they transmit, and more broadly, dengue transmission cycles. Higher temperatures increase the rate of larval development and shorten the emergence of adult mosquitoes, increase the biting rate of mosquito and reduce the time required for virus replication within the mosquito. Extreme higher temperatures may reduce mosquito survival time, which could offset the positive effect on mosquito abundance [69]. Evidence had accrued to show the link between temperature and dengue incidence rates [9,34,36,47]. These studies have used a range of statistical approaches considering different temperature parameters (e.g. mean, maximum and minimum temperatures), and the results are generally consistent, indicating that the epidemics of dengue are driven by climate to some extent.

Relative humidity is another key factor that influences mosquitoes’ life cycle at different stages. The combined effect of temperature and humidity significantly influences the number of blood meals and can also affect the survival rate of the vector, and the probability that it will become infected and able to transmit dengue [70]. In the literature, relative humidity and temperature are the two most important variables with potential impact on dengue transmission. Vapour pressure or relative humidity is affected by a combination of rainfall and temperature and influences the mosquito lifespan and thus the potential for transmission of the virus. Hales et al. found that annual average vapour pressure was the most important climatic predictor of global dengue occurrence [33]. Therefore, temperature, rainfall and relative humidity are important determinants of the geographic limits within which dengue transmission can be expected to continue, primarily through their effects on the *Aedes* vector. Furthermore, within areas where minimum thresholds of these climate parameters are sufficient to maintain dengue transmission, seasonal fluctuations in these parameters will be important determinants of the duration and potentially the intensity of transmission.

Several studies have shown that the increased temperatures and relative humidity are determining factors in predicting changes of the dengue transmission [31,32]. Contrasting, rainfall did not appear to play a significant role because many breeding sites of *Ae. aegypti* were more dependent on human behaviour than on rainfall for their development and survival [71-73]. This might partly explain the lower impact of rainfall compared to other climatic variables on the dengue incidence. However, Chaves et al. [74] suggested that a series of rainfall followed by low/ lack of rainfall could intimidate sharp decrease in mosquito populations, for example, in Puerto Rico.

Dengue transmission in endemic settings is characterised by non-linear dynamics, with strong seasonality, multi-annual oscillations and non-stationary temporal variations. Seasonal and multi-annual cycles in dengue incidence vary over time and space. Besides the seasonality of dengue transmission, periodic epidemics and more irregular intervals of outbreaks are commonly observed [34,75-77].

Evidence suggests that inter-annual and seasonal climate variability have a direct influence on the transmission of dengue [17,32,34,41,78,79]. This evidence has been assessed at the country level in order to determine the possible consequences of the expected future climate change [33,38]. These studies have highlighted that many climatic variables play a key role in dengue transmission as discussed above.

Many studies have highlighted the importance of lag time, at monthly scales. For example, in Taiwan, there was a significant positive correlation with the maximum temperature at lag 1–4 months, the minimum temperature at lag 1–3 months and the relative humidity at lag 1–3 months [49]. In New Caledonia, significant associations were found between temperature and dengue transmission at lag-0 [31].

The delayed effect (or time lag) of climatic variables on dengue incidence could be explained by climatic factors which do not directly influence the incidence of dengue but do so indirectly. This is through their effect on the life-cycle dynamics of both vector and virus. This starts with mosquito hatching, larval and pupal development, adult emergence and virus amplification, incubation in humans culminating in a dengue outbreak and results in a cumulative time lag [36,70]. Depending on the respective lag between the biological cycle or mosquito life-stage and the clinical symptoms, the lag between climate data and incidence data will differ. The lag is expected to be shorter for minimum temperatures that are usually associated with adult mosquito’s mortality, longer for high relative humidity, both related to adult survival and hatching. On the other side, the mean temperature is involved in all biological cycles of *Ae. aegypti* that take more time to influence the dengue incidence [5,36,78].

### Strengths and limitations of studies

Several statistical analytical methods have been used to determine the relationship between climate variables (and climate change) and dengue, including cross correlations, Poisson, logistic and multivariate regression, SARIMA-time series and wavelet time series (Table 1). Many have been successful [31,34,41,49,50] in establishing climate and dengue relationships and developing predictive models of dengue based on climate relationships. Minimum, maximum and mean temperatures, relative humidity and rainfall were the most important climate variables that predict the dengue. However, these variables are predictive at specific lags of time.

Remarkably, due to data constraints, modelling analyses were conducted using aggregated data over large spatial scales or long time periods [34]. Studies based on long time scales and large geographic areas may be unsuccessful in describing the influences that happen over daily or weekly periods and climate changes that occur at country level [45].

In general, the predictive power and model robustness would be better improved with large data over longer periods. For example, Gharbi et al. [36] developed statistical predictive models that were build-up on <10 years of data and could only be validated over a 1-year period. Hence, it is difficult to say whether the relationships they found will restrain in time.

The dengue cases/incidence reported could be over- and under reported. These may change over time and geographical area. Additionally, the reported dengue cases may be influenced by case definitions, availability of public health systems and subclinical cases documented.

Therefore, it is important to consider all these factors before identifying the relationships between climate and dengue disease transmission. Patz et al. [38] provided a good framework for future research on climate change and dengue transmission. However, these estimates should be updated based on better improved resolution with current GCM projections.

### Recommendations for future research

We recommend the following five directions for future research: 1) Disease surveillance need to be improved for effective dengue prevention and control programs. The new approach to surveillance lays emphasis on the inter-epidemic period, as information on the dengue endemicity is important in predicting dengue epidemics. The surveillance of active dengue virus activity during inter-epidemic periods provides information on prevalent virus serotypes in the area. Any subsequent introduction of another serotype would then require control measures to prevent the increase in transmission of the virus, thereby helping in containing the impending epidemic and reducing the incidence of dengue. 2) Better understanding of dengue ecology is required to predict the climate-biological relationships on dengue transmission. 3) Application of advanced spatio-temporal modelling approaches in dengue research is required to more fully understand the complex relationship between climate and dengue and thereby obtain better prediction. Moreover, these approaches, or at least the outcomes of these models need to be better integrated. 4) Uncertainties due to confounding effects of urbanisation, population growth and tourism development are required to develop scenarios based on future projections of population growth and socio-economic development, including human behaviour. 5) There is a clear need for inter-disciplinary collaborations with ecologists, sociologists, micro-biologists, bio-statisticians and epidemiologists. Two areas need special attention: one is in the area of climate modelling to address issues of spatial and temporal scale and analytical methods, and the other relates to dengue incidence data quality control with the reporting agency (e.g., laboratories, hospitals, health centres) addressing issues such as underreporting and misdiagnosis, dengue case definition, clinical or lab-confirmed diagnosis, inpatient and outs reporting, specific ages and/or dengue severity reported. From an analytical and modelling perspective, analyses need to be able to consider localised long-term time series demographic, socio-economic and environmental conditions.

Finally, we recommend that caution should be taken when estimating the relationships between climate variables (and climate change) and dengue in the following aspects: use of time lags, the analysis of extreme climatic events, the differences between seasonal and long term trends, nonlinear effects and threshold effects in the associations. In addition, more emphasis should be given to data quality and the use of information for decision-making.

## Conclusion

The weight of evidence about climate change impacts on dengue indicates that dengue transmission is sensitive to climate variability and change. We believe that it is important to develop, employ and integrate different quantitative modelling approaches for dengue transmission compatible with long-term data on climate and other socio-ecological changes and this would advance projections of the impact of climate on dengue transmission.

## Competing interests

The authors declare that they have no competing interests.

## Authors’ contributions

SN participated in data extraction, analysis and drafted the manuscript. PD, JM, JB, KM and ST critically revised the manuscript. All authors read and approved the manuscript.

## Pre-publication history

The pre-publication history for this paper can be accessed here:

http://www.biomedcentral.com/1471-2334/14/167/prepub
